# Risk Factors for Transport-Related Problem Behaviors in Horses: A New Zealand Survey

**DOI:** 10.3390/ani8080134

**Published:** 2018-08-02

**Authors:** Barbara Padalino, Chris W. Rogers, Danielle Guiver, Janis P. Bridges, Christopher B. Riley

**Affiliations:** 1 College of Veterinary Medicine and Life Sciences, City University of Hong Kong, Kowloon, Hong Kong, China; 2 Department of Veterinary Medicine, University of Bari, 70010 Bari, Italy; 3 School of Veterinary Science, Massey University, Palmerston North 4470, New Zealand; C.W.Rogers@massey.ac.nz (C.W.R.); Danielle.guiver@gmail.com (D.G.); J.P.Bridges@massey.ac.nz (J.P.B.); C.B.Riley@massey.ac.nz (C.B.R.)

**Keywords:** problem behavior, transport, training, horse

## Abstract

**Simple Summary:**

Transport-related problem behaviors (TRPBs) are unwanted behaviors exhibited by horses in one or multiple phases of transport contributing to the injury of the horses and their handlers. The aim of this study was to identify risk factors for TRPBs in support of the development of best practices that minimize their incidence, and safeguard horse and handler wellbeing. An online cross-sectional survey was designed and disseminated to New Zealand equine industry members. Respondents were asked whether one of their horses had shown TRPBs during the two previous years, and to describe their equine background and experience, the method or way in which they had trained their horses for loading and travelling, the type of aids they used for loading and the type of vehicle. At least one horse was reported as showing a TRPB by almost one out of four of respondents. The type of vehicle, of training and of aids used for loading and travelling resulted associated with TRPBs. These findings may be useful to enhance horse welfare by educating people in charge of moving horses on appropriate training methods and vehicle selection for road transportation.

**Abstract:**

Transport-related problem behaviors (TRPBs) are common in horses and can cause injury to both the horses and their handlers. This study aimed to identify possible risk factors for TRPBs to inform approaches to mitigate TRPBs incidence and enhance horse welfare. An online cross-sectional survey was conducted to explore the prevalence of TRPBs and their association with human-, training- and transport management-related factors in New Zealand. The survey generated 1124 valid responses that were analyzed using descriptive statistics, and logistic regression analyses. Having at least one horse with TRPB was reported by 249/1124 (22.2%) respondents during the two previous years. Of these, 21/249 (8.4%) occurred during pre-loading, 78/249 (31.3%) during loading, 132/249 (53.0%) while travelling, and 18/249 (7.3%) during unloading. Our findings indicate that the use of negative reinforcement and positive punishment as training methods, using a whip or food for loading, and travelling in a straight load trailer/float while offering food were associated with a higher likelihood of TRPBs. Cross-sectional studies cannot determine causality and findings should be interpreted with caution, and evaluated in further experimental studies. The authors suggest that education on appropriate training methods for transport, and vehicle selection may mitigate the risk for TRPBs in horses.

## 1. Introduction

Transport-related problem behaviors (TRPBs) are unwanted behaviors exhibited by horses in one or multiple phases of transport [1]. TRPBs have been categorized as associated with pre-loading (e.g., increased vocalizations and pawing), loading (e.g., refusal to load), travelling (e.g., repeated kicking of parts of the vehicle or other horses) and unloading (e.g., rushing off the ramp) [2]. Refusal to load and scrambling (i.e., loss of footing and repeated attempts to keep from falling) en route are the most commonly reported within the literature [3,4,5]. TRPBs often frustrate horse handlers due to time delays, or inability to transport their horse to the sport event or leisure activity [1]. Injuries to the horse and handler are often reported with TRPBs, compromising welfare and safety [2]. TRPBs and their consequences are apparently common. A recent YouTube search produced more than 67,000 videos related to TRPBs and their possible solutions; many touted solutions did not comply with published evidence-based recommendations [1].

Scientifically based training methods have been studied since the 80s and are recommended to mitigate the incidence of TRPBs and their consequences [1,6]. Habituation to loading and travelling is one approach to desensitizing horses to their innate fear of transport vehicles [6]. The technique commences with a series of simulations of loading, staying inside the transport vehicle and unloading until the horse no longer shows anxiety. Short journeys are then undertaken, initially within the property, and then the duration is progressively increased. This training should be carried out before the first real journey. Ideally habituation for transport should start with the foal, following the mare into the truck or trailer. Another method to habituate or desensitize the horse is to leave a transport vehicle in the paddock and to feed the horse inside it [3]. To minimize TRPBs developing in frequent travellers resulting from an association with performance activities, horses should not only experience journeys to official competition, but also to low stress destinations (e.g., pasture) [3]. With the successful implementation of the afore mentioned techniques, loading for transport and travelling should be less stressful for the horse, minimizing the occurrence of TRPBs [7].

Self-loading has also been suggested to train horses to load freely and prevent TRPBs [8]. As per the habituation training, different methods may be used to reach the goal of self-loading and should be performed before a required journey. Frequently suggested methods to teach a horse to self-load are clicker and target training (based on positive reinforcement) [9,10], but other training methods (based on negative reinforcement) are also used [11]. Self-loading requires patience and a combination of operant and classical conditioning that results in a horse that self-loads on command. The cues can be acoustic or visual, such as seeing the open ramp or positioning of the lead rope on the neck [2]. Habituation and self-loading have been found to be associated with lower levels of reported TRPBs, but their use is rarely reported within the literature [1,2,12]. Despite the scientific evidence of describing appropriate methods for training horses [1,2,13], many people may not train their horses for loading and travelling in transport vehicles, or may use inappropriate training methods based solely on negative reinforcement or positive punishment [2,8,12].

In a survey on horse transport management and issues in Australia 38% of respondents reported having at least one horse with TRPBs [14]. Most did not train their horses for loading and travelling, and TRPBs were associated with training methods, forward-facing trailers, and the use of whip and bum (butt) ropes for loading [2]. A survey on TRPBs has not previously been conducted in New Zealand, and associations with transport management and human-related factors have not been reported. The authors hypothesized that TRPBs were associated with the experience of respondents in horse handling, the type of training for loading and travelling, and transport management factors. The study aimed to identify risk factors for TRPBs in support of the development of best practices that minimize their incidence, and safeguard horse and handler wellbeing.

## 2. Materials and Methods

This online survey-based study was approved by the Massey University Human Ethics Committee as low risk (Ethics Notification Number: 4000017178).

### 2.1. Target Population

The target population for the survey were New Zealand equine industry participants. Eligible respondents were required to have one or more horses in their care and to be responsible for organizing transport of horses for professional or recreational purposes at least once during the two years prior to completing the survey. For inclusion in this study, the respondent had to respond to the question related to TRPBs (Q.18, Supplementary file). The approximate size of this population was estimated to be 90,000 [15,16]. 1053 surveys were therefore required to attain a 95% confidence level, and an error level of ±5% [17].

### 2.2. Study Design and Data Collection

A cross-sectional online survey was conducted in New Zealand between 7 February 2017 and 16 May 2017 (about 3 months). The survey was designed taking into account the findings of a survey on transport-related issues and practices recently distributed in Australia [14]. Key survey design features to ensure valid results were considered including a process of iterative review [18,19]. The survey was digitized (Qualtrics, New Zealand) and piloted among six horse owning staff members at Massey University. An invitation letter and the link to the online survey were provided to a wide range of New Zealand horse sports and organizations, and the survey link was also published on equestrian websites related to those organizations. The link was also promoted through a national horse magazine, relevant social media pages and online horse forums [20]. The survey exploring TRPBs contained 18 closed and one open questions (Supplementary file). There were questions about the respondents (gender, equine industry sector in which they were involved; amateur or professional relationship with the horse, experience with horse handling, ability to identify equine distress, number of horses in care), the journey (frequency, duration), transport management (type of vehicle, positioning inside the vehicle, use of sedation and protective equipment pre-journey, type of restraint during transport, presence of food *en route*), the use of a specific training for loading and travelling, the use of equipment for loading (whip, bum (butt)-rope, food), and experience of a horse showing TRPB (and in what phase of transport).

### 2.3. Statistical Analysis

The contributions of the explanatory variables related to the respondents, the journey and the transport management characteristics (Table 1) were evaluated with respect to the occurrence of TRPBs. To ensure a more balanced dataset (i.e., no category with less than 5% of the population of values), data from the Thoroughbred and Standardbred racing respondents were combined into the same category, and those reporting “some” or “moderate” ability to assess distress in horses were combined into the same group for analyses.

Respondents were asked to describe the method or way in which they had trained their horses for loading and travelling. The replies were classified by one of the researchers (BP) with expertise in animal training into the following training method categories: habituation (H), self-loading (SL), no identified training (NT), operant conditioning with a combination of negative reinforcement and positive punishment (R−P+), and operant conditioning using positive reinforcement (R+) (Table 2) [1]. To be considered as habituation, the respondent had to have specified that the training was applied before a real trip, and repeated several times with the aim to desensitize/get the horses used to the transport procedures. To be considered as SL, the respondent had to have written “self-loading”.

Based on the respondents’ response to the multiple-choice question on the equipment used to load horses, the equipment used was classified into the following categories: food (yes, no), whip (yes, no), bum rope (yes, no). Some respondents reported using a stallion bit (91/1133), bridle (25/1133), load ‘n’ tie (10/1133; a proprietary rope system placed around the body of the horse and connected to a lead rope separate from that on the halter), towel (1/1133), or lasso (1/1133), but the frequency of responses for these categories were insufficient for analyses of their possible effects on the behavioral outcome (TRPBs).

Reporting the observation of TRPBs (i.e., if the horse had shown a problem behavior) was used as the binary outcome variable (yes/no). Based on the results of the survey question addressing the outcome variable, TRPBs were classified into: pre-loading (PLPB), loading (LPB), travelling (TPB) and unloading (UPB).

The initial descriptive analysis included the creation of frequency tables and was conducted using Statulor β (http://statulator.com/descriptive.html). The statistical software R (R v 3.4.1, 2017 Foundation for Statistical Computing, Vienna, Austria) was used for univariate and multivariable logistic regression analyses.

A model was derived for TRPBs; the outcome was binary (1/0, behavioral problem/non- behavioral problem). The explanatory variables explored included gender, industry sector, involvement, experience, number of horses, distress, journey frequency, journey distance, vehicle, direction of travel of the horse within the vehicle, sedation, protective equipment, rug, boots, type of training, whip, food, restrain and food *en route*. Initially univariate logistic regression was performed, and *P* values were calculated using Wald Test. Each predictor variable returning a *p* < 0.20 from the univariate modelling was considered for inclusion in a multivariable model (Table S1). A step-wise backward elimination procedure was then conducted whereby predictive variables were removed until all variables in the final model had a Wald’s *p* < 0.05 indicating significance. A set of basic diagnostic statistics including fitted and standardized residuals and leverage was examined for adherence to model assumptions. No influential points were detected. The models were compared using ANOVA of deviance function in R, and the model with the lowest Akaike information criterion (AIC) was chosen [24]. The findings are presented as odds ratios (OR) and confidence intervals (95%CI) for each predictive variable.

## 3. Results

### 3.1. Survey Response

There were 1486 initial logins to the survey, but only 1124 (75.6%) respondents answered the question on the presence or absence of TRPBs, and were therefore considered valid for data analyses. This sample size resulted in a 95% confidence level, and an error level of ± 2.8% for the study population.

### 3.2. Descriptive Statistics of The Predictive and Outcome Variables

Table 3 is a frequency table showing the results of the survey questions. In the two years before the survey, at least one horse was reported as showing a TRPB by 249/1124 (22.2%, 95%CI: 19.7–24.6%) of respondents. Of these 249 reported TRPBs, 21/249 (8.4%, 95%CI: 4.9–11.8%) were described as occurring during pre-loading, 78/249 (31.3%, 95%CI: 25.5–37.1%) loading, 132/249 (53.0%, 95%CI: 46.8–59.1%) while travelling, and 18/249 (7.3%, 95%CI: 4.1–10.5%) on unloading.

### 3.3. Univariate and Multivariable Logistic Regression

The results of the univariate logistic regression analyses are presented in Table 4. Respondents with less than 5 years of experience in horse handling, and those claiming a high ability to identify distress in horses were more likely to report a horse with TRPBs. Higher odds of TRPBs was found for horses transported in straight load trailers/floats and by commercial trucking companies, those trained using R-P+, loaded using a whip or food, restrained using a long rope, or fed *en route*. 

The results of the multivariable logistic regression analyses (χ^2^ = 60.3, df = 11, *p* < 0.001) are reported in Table 5. Horses transported in a straight load float/trailer, a large truck, or using a commercial company were at higher risk of showing TRPBs in comparison with horses transported in small trucks (2–3 horses). Compared with habituation, the only training method which increased the risk of TRPBs was operant conditioning with the use of negative reinforcement and positive punishment (R−P+). The use of whip and food during loading and the practice of feeding the horse en route were also associated with higher odds of TRPBs.

## 4. Discussion

This cross-sectional study documented the prevalence of TRPBs in horses transported in New Zealand for pleasure or professional purposes, and for the first time explored associations between TRPBs, and human- and transport management- factors. This study is the first to identify a relationship between TRPBs and management-related factors such as horse handling experience, the ability to recognize distress in horse, appropriate transport-related training methods, the use of whip during loading, the type of restraint used in transit, and the use of feeding during loading and *en route*. These data support the authors’ hypotheses. However, the findings of surveys rely upon the retrospective recollection of participants, and should not be construed as defining causal relationships among factors [25]. It is worth highlighting that the detailed nature of the relationships identified between TRPBs and transport management routines in this study cannot be precisely defined. The latter is a common limitation of all studies exploring management factors and based on survey methods [25,26]. For instance, management practices may have been applied in attempts to minimize TRPBs rather than being causative factors. For example, the associations between TRPBs and the practices of feeding and restraint with a long rope en route are more likely a reflection of owners’ attempts to implement better practices, and require empirical further research. 

Our findings documented characteristics of people involved in the care and transport of horses in New Zealand and their transport practices. In agreement with other studies, most of our respondents were women taking care of fewer than five horses in their property, involved with horses as amateur mainly in equestrian sports or pleasure activities, moving horses frequently and on short distance [27,28,29]. As described in Australia, the transport vehicle most frequently used was a two-horse straight load trailer (float) with the horse facing in a forward direction of travel, and boots were the most frequently used protective equipment [14]. Even though wearing a rug, cross-tying or tying on a short rope en route have been discouraged as inconsistent with best practice [30], one third of the respondents applied these practices. On the other hand, it is worth highlighting that New Zealanders associated with horse transport appear (based on the survey data) to understand the importance of training horses for loading and travelling [7]. The data suggest that New Zealand respondents more commonly train their horses than Australians involved in horse transport (80% versus 40%) [14]. In particular, habituation and positive reinforcement training were more often used by horse owners/trainers in New Zealand compared to horse owners/trainers in Australia [2]. However, whips and bum ropes were more frequently used as aid to load horses than reported in Australia [2]. In New Zealand one out of three respondents documented feeding their horses *en route*. This practice was more frequently applied in Australia [14] and may be related to the longer distances generally travelled by horses in Australia. Whether feeding en route is or is not best practice, it is still a matter of debate within the literature. 

Overall, the rate of TRPBs was lower in New Zealand than in Australia (38.6%) [14]. In contrast with the literature, New Zealand respondents recalled having more TRPBs in transit than when loading horses; loading has been associated more frequently with TRPBs in many other studies [1,2,4]. These differences may reflect the populations of respondents with respect to their demographic, cultural backgrounds and perception of TRPBs. It may also lie in the type of journey, the different transport management practices and particularly in the different training practices used. Most of our respondents used training methods, such as SL or operant conditioning, focused on teaching horses how to load. This may explain why the prevalence of loading TRPBs was less common than those encountered in transit. 

The Australian, New Zealand and European codes of animal transport [31,32,33] recommend that horses should be loaded by people with proven experience in horse handling. A course on how to identify and manage stress in the transported animals is mandatory in Europe for any commercial journey [31], but not in New Zealand. This study is the first to confirm an association between less experience (particularly less than five years) in horse handling, and less ability to identify horse distress with higher odds of TRPBs, adding scientific evidence for the recommendations contained within these animal transport codes. While TRPBs have been associated with a higher risk of injury to horses [2], training to increase horse handling skills and understanding of horse behavior is recommended to minimize the incidence of TRPBs and their consequences in New Zealand.

The findings described in the current report demonstrate an association of TRPBs with straight load trailers, confirming results reported in a previous study [2]. Loading in a small and dark two-horse straight load trailer has been described as an unpleasant and frightening experience for any horse, taking into account the equine field of vision [13]. Straight load trailers are less wide than angle or backward facing loading trailers, which have been suggested to be less frightening and easier to load horses into [34]. The width of space allocated to the horses in transit may affect their stress responses. Stull et al. [35] demonstrated that rectal temperature, white blood cell count, cortisol level and neutrophil-lymphocytes ratio were higher in horses travelling in straight deck trailers (1.14 m^2^) than in pot-belly trailers (1.31 m^2^). Furthermore, as both loading and travelling problems were considered in our dataset, one reason for the association may be because a forward-facing position was found to be less preferred and unstable in comparison with sideways and backward travel [36,37,38]. Consequently, travelling in a straight load trailer may be more likely to cause scrambling and other TRPBs. The authors also found an association with large trucks and with the movement of horses in commercial company trucks. This finding is new and requires further exploration. Factors for consideration may include the effect of the angle of the loading ramp, often steeper for these types of trucks, and the effect of loading into a new environment where there are more likely to be non-familiar horses that were already loaded at other points of departure. Although further studies are required, it is possible that horses transported in these types of vehicles are more likely to encounter non-familiar horses, increasing the risk of social stress on horses, and causing more aggressive behavior (biting or kicking other horses) and other problem behaviors in transit which were more frequently reported in comparison to loading problems by the respondents in the current study. Horses transported in commercial trucks spend the first hour of their journey sniffing and exploring the new environment, reporting the highest frequency of stress-related behaviors in the first hour, and showing aggressive behaviors toward non-familiar neighbors in the last part of the journey [39]. It is possible that these stress-related and aggressive behaviors were interpreted and reported as TRPBs by our respondents. The use of video cameras to monitor behavior during commercial transportation is mandatory in Europe and is recommended in commercial and non-commercial transportation to clarify the nature of travelling problem behaviors, and provided indications to minimize their consequences. In European commercial transportation, horses are only allowed to be transported in vehicles equipped with solid dividers that go all the way to the ground. In our survey there were no questions about the type of dividers and other features of trailer design. Further studies are needed to explore the possible associations between the features of trailer design and TRPBs.

This manuscript provides evidence confirming that the use of training based on negative reinforcement and positive punishment is not consistent with best practice for teaching horses to load and travel [1]. In the case of a horse that refuses to load, the use of operant conditioning based on negative reinforcement (i.e., the release of the pressure) often fails. This is because the pressure is frequently released when the horse forcefully moves away from the ramp, overcoming the strength of the human handler [2]. Therefore, negative reinforcement should not be used because in this situation the horse learns that it can “win the battle pressure against pressure” [13]. The use of positive punishment such as whipping has been already described by Houpt (1982) as a method which can momentarily solve the problem (i.e., the horse loads to avoid whipping), but creates a negative association (i.e., trailer loading equals being whipped). Subsequently, the next time transport is attempted, the horse misbehaves at the view of the trailer, increasing the odds of TRPBs at loading. Whipping at loading may also increase the risk of TRPBs in transit, because the horse loads with a higher level of fear and anxiety, and starts to show TRPBs while the vehicle is moving [1]. The findings of the current study agree with the literature, with the use of a whip at loading increasing twofold the likelihood of TRPBs being reported. Food has been described as a tool for positive reinforcement recommended to retrain horses with loading problems [10]. In contrast, for the current study the use of food during loading was associated with a higher likelihood of TRPBs being reported. There are several possible explanations for this finding. A first consideration is that positive reinforcement has been shown to be less effective in the presence of stressors, because learning performance is impaired [40]. Secondly, it is possible that respondents may have used food inappropriately to motivate the horse to load rather than as a proper reward given in response to the wanted behavior [41]. Finally, it is possible that respondents used food at loading in attempts to minimize TRPBs; positive reinforcement may have been indeed given to treat TRPBs rather than causing them. As mentioned above, the nature of the associations found using this type of surveys cannot precisely defined. A limitation of the current study is that training with respect to TRPBs was considered without distinguishing among pre-loading, loading, travelling and unloading problem behaviors as was previously described by Padalino et al. [2]. The latter authors reported that habituation was the only method which minimized the risk of any type of TRPBs, self-loading decreased only TRPBs at loading and unloading and operant conditioning increased all type of TRPBs. The current study suggests that negative reinforcement and positive punishment, particularly using a whip, are more likely to increase the prevalence of TRPBs and are not recommended to train horses for loading and travelling. However, a prospective study with a more critical evaluation of the effects of different types of training on the prevention or management of TRPBs would be of value.

In the current study feeding while travelling, and the use of a long rope for restraint were associated in the univariate regression with an increased likelihood of TRPBs. The effects of the feeding and restraint en route on the risk of transport-related respiratory disease have been evaluated in previous studies [42,43,44,45]. No restraint or the use of a long rope, and positioning food at least at the knee level have been recommended to mitigate the risk of transport pneumonia [41,42,43,44]. The effects of the type of restraint and feeding en route on TRPBs have not previously been described in the literature. Many horse owners believe that offering food en route calms horses, and less restraint allows the horse to meet postural corrections when the vehicle is slowing down [34]. These practices could have therefore used in the attempt to reduce the incidence of TRPBs and cannot be interpreted as a cause of TRPBs, as afore mentioned [26]. Overall, our findings should be considered preliminary and the effects of travelling with the presence of food, its position and type should also be tested in the future to clarify their effects on transport-related health and behavioral problems.

In the final multivariable model, the type of vehicle used for horse transport, the training used for loading and travelling, the use of whip and food during loading, and the presence of food en route remained as the most significant factors associated with TRPBs. These findings highlighted the importance of educating people in charge of moving horses to read horse body language, and understand animal learning principles (which are the basis of a correct horse handling in equitation science) [22,40]. Unfortunately, there are no evidence-based design standards for the design of horse trailers used for non-commercial transport in New Zealand and most other countries [34]. More research is needed to better understand what type of vehicle design (e.g., bay space, positioning, lights) may decrease transport stress and TRPBs in horses.

The findings from the study should be interpreted with caution for the reasons outlined above. Although survey respondents were asked about initial training for loading and travelling, it was difficult to differentiate whether the respondents reported their initial training for transport or the retraining methods used to treat horses showing TRPBs. The author suggests that our findings about the training methods should not be applied to horses suffering from established TRPBs, and that these animals undergo an appropriate retraining plan as suggested in the literature [8,10]. Notwithstanding the limitations of the survey, this study was useful for identifying human, management and training factors which were associated with TRPBs and for providing a basis for the development of prospective studies. The application of these findings and subsequent research may be useful for enhancing the wellbeing and safety of the horse and its handler during transport.

## 5. Conclusions

This study is the first to identify associations between TRPBs and human-, training- and transport management factors in New Zealand. Among human factors, experience in horse handling and the ability of the horse handler to recognize distress were associated with lower odds of TRPBs. Regarding training for loading and travelling, habituation was confirmed as the preferred method to minimize the prevalence of TRPBs. The two-horse straight load trailer was confirmed as the transport vehicle most likely to be associated with a higher likelihood of TRPBs. The other associations found need to be confirmed with further studies. The findings of this study add to the knowledge base that can be used to educate people involved in horse transportation, to decrease the risk of TRPBs, to safeguard horse welfare and safety of the horse handler during transport.

## Figures and Tables

**Table 1:** Classification of the survey variables.

Name	Description	Values
**Respondent characteristics**		
Gender	Gender of the respondent	Male, Female
Sector	Sector of the horse industry in which the respondent was involved	Thoroughbred or Standardbred racing, Dressage, Eventing, Show Jumping, Pony Club, Endurance and Competition Trail Riding, Horse breeding, Recreational non-competitive, Other (i.e., Hunting, Western, Polo, Showing)
Involvement	Nature of the respondent’s involvement with horses	Professional (involved with horses for financial reward), Amateur (involved with horses as a hobby or recreationally)
Experience	Respondent’s years of experience handling horses	1–5, 6–10, 11–20, 21–30, 31–40, 41–50, >51
Number of horses	Number of horses kept with their horse described in the survey	1–2, 3–5, 6–10, 11–15, 16–30, >31
Distress	Respondent’s self-assessment of their own ability to identify a horse in distress	1—none, 2—some or 3—moderate, 4—high, 5—very high
**Journey characteristics**		
Journey frequency	Frequency of organized transport events	Daily, 2–5 times a week, once a week, fortnightly, monthly, <once a month
Journey distance	Average journey distance (km)	1–30, 31–60, 61–90, 91–120, 120–240, >241
**Transport management characteristics**		
Vehicle	Transport vehicle usually used for moving horses	Small truck—2 to 3 horses, Large truck—more than 3 horses, Gooseneck, Float/trailer—angle load, Float/trailer—straight load, Use of a commercial trucking company
Direction	Direction horse is facing during travel	Head facing or angled to the front, Head facing or angled to the rear, Horse free and unrestrained
Sedation	Use of sedation or other products to calm the horse(s) prior to transportation	Yes, No
Protective equipment	Use of one or more items of protective equipment (Leg boots, leg bandage, pool protector, Tail guard, Neck/Body rug, other)	Yes, No
Rugs	The use of rug	Yes, No
Boots	The use of boots	Yes, No
Training	If and how the respondent trained the horse to load, travel and unload	Habituation, Self-loading, operant conditioning R− P+ ^1^, R+ ^2^, no identified training method
Whip	The respondent’s use of a whip during loading procedure	Yes, No
Bum rope	The respondent’s use of a bum rope during loading procedure	Yes, No
Food	The respondent’s use of food during loading procedure	Yes, No
Restraint	How the respondent restrained the horse *en route*	I do not restrain my horse; Tie up on a short rope; Tie up on a long rope; Cross tie
Food *en route*	Did the respondent offer food to the horse(s) when travelling	Yes, No

^1^ R−P+: operant conditioning with a combination of negative reinforcement and positive punishment; ^2^ R+: operant conditioning using positive reinforcement.

**Table 2:** Definitions of training categories as modified by Padalino et al. (2017) and examples of respondents’ replies for the training category.

Training Category	Definition	Examples of Typical Responses to the Question: “Have You Use Any Training to Aid in Transporting Your Horses? If So, Describe the Training Tool (i.e., Training in Loading and Unloading the Vehicle)”
Habituation (H)	The habituation category included techniques used to habituate horses to all aspects of transport prior to travel, such as familiarizing young (foals and weanlings) and new horses to the transport vehicle, repeated loading and unloading prior to travel, and/or taking the horses on short trips, and/or using an experienced companion for short trips prior to undertaking longer journeys for specific purposes [7].	1. Leaving the float in a paddock with food and water inside. 2. Using food as a reinforcement, gradually encouraging the horse to go into the float and get used to partitions/bars/ramp, and put in place before short drives around the block. 3. Getting them used to walking in and out of a float and standing calmly with another horse in the float. 4. Educating them to load from young age, load but don’t travel initially, small trips to begin with monitoring how they are travelling, take a mate with them to begin with, remove partition in float to start with, feed reward for loading initially, drive very slowly to begin with; do everything possible to ensure the first few experiences in travelling are good ones. 5. Repetition of loading and unloading and then when good, increasing time spent in moving truck.
Self-loading (SL)	Operant conditioning and classical conditioning leading to the horse self-loading onto the vehicle on a verbal, visual or other classically conditioned cue [8,9,21].	1. Taught self-loading as usually loading by myself. 2. Self-loads and unloads. 3. Have trained horses to self-load and know when they are allowed to come off the float. 4. All horses are taught to self-load, stand, have the ramp up and be clipped up with and without a companion experienced traveller before taking them on a journey.
No identified training (NT)	Respondents did not train their horse to load or travel. Their horses had already been educated with no identified method.	No. When I bought my horses they had already been trained. They travel regularly now.
Operant conditioning with a combination of negative reinforcement and positive punishment (R−P+)	Negative reinforcement (release of the pressure at the time of the wanted behavior) or positive punishment (adding an unpleasant stimulus (whipping, or applying pressure with the bum rope) at the unwanted behavior) [22,23].	1. Pressure and release. 2. Natural horsemanship pressure and release. 3. Use of a whip to encourage forward movement. 4. Tapping on sides. 5. Andrew McLean method of pressure and release to go forward onto float/truck [23]. 6. Bum ropes. 7. Tap front legs initially to teach loading. 8. Use a bum rope if required, walk them up lifting a leg at a time. 9. Andrew Mclean method—pressure and release with whip tapping [23]. 10. Reward (release of the bum rope pressure) with doing the right thing. 11. Whatever necessary (bum rope, stallion bit, whip). 12. Andrew Mclean method of loading until horse is comfortable standing in float. There really isn't much information about how to train them for the travelling bit—they really have to learn that on the journey
Operant conditioning with use of positive reinforcement (R+)	Rewarding the wanted behavior using food or other pleasant reinforcement [10,22].	1. Equitation science, positive reinforcement. 2. Slowly and consistent with lots of positive reinforcement. 3. Slowly walking on, rewarding each step with food, horses learn it is a safe happy place. 4. Trained via positive reinforcement with feed to load (very good orientated). 5. Positive reinforcement. 6. R+.

**Table 3:** Frequency table of the responses to a survey on horse road transport-related problems behaviors in New Zealand

Variable Name	Category	Count	Percent	95%CI
Gender	Female	943	84.6	82.4–86.7
	Male	171	15.4	13.2–17.5
	Total	1114	100	
	Missing Values	10	0.9	
Sector	Dressage	130	11.6	9.7–13.4
	Endurance and Competitive trail riding	54	4.8	3.5–6.0
	Eventing	119	10.6	8.8–12.4
	Horse breeding	67	6.0	4.6–7.4
	Other	96	8.5	6.8–10.1
	Pony club	78	6.9	5.4–8.3
	Racing (Thoroughbred and Standardbred)	238	21.2	18.8–23.6
	Recreational riding	208	18.5	16.2–20.7
	Show jumping	134	11.9	10.0–13.8
	Total	1124	100	
Involvement	Amateur	840	75.3	72.7–77.8
	Professional	275	24.7	22.1–27.2
	Total	1115	100	
	Missing Values	9	0.8	
Experience	1–5	47	4.2	3.0–5.3
	6–10	95	8.5	6.8–10.1
	11–20	268	24.0	21.4–26.5
	21–30	228	20.5	18.2–22.8
	31–40	258	23.2	20.7–25.6
	41–50	149	13.4	11.3–15.4
	>51	69	6.2	4.7–7.6
	Total	1114	100	
	Missing Values	10	0.9	
Number of horses	1–2	196	17.7	15.4–19.9
	3–5	356	32.1	29.3–34.8
	6–10	232	20.9	18.5–23.2
	11–15	96	8.6	6.9–10.2
	16–30	133	12.0	10.1–13.9
	>31	97	8.7	7.0–10.3
	Total	1110	100	
	Missing Values	14	1.2	
Distress	5—very high	587	52.3	49.3–55.2
	4—high	463	41.2	38.3–44.1
	2—some or 3—moderate	73	6.5	5.0–7.9
	1—none	0	0	
	Total	1123	100	
	Missing Values	1	0.1	
Journey frequency	Daily	79	7.0	5.5–8.4
	2 to 5 times a week	280	24.9	22.4–27.4
	Once weekly	275	24.5	21.9–27.0
	Fortnightly	209	18.6	16.3–20.9
	Monthly	127	11.3	9.4–13.1
	Less than once a month	154	13.7	11.6–15.7
	Total	1124	100	
Journey distance (min)	1–30	140	12.9	10.9–14.9
	31–60	315	29.1	26.3–31.8
	61–90	98	9.0	7.2–10.7
	91–120	285	26.3	23.6–28.9
	120–240	81	7.5	5.9–9.0
	>241	165	15.2	13.0–17.3
	Total	1084	100	
	Missing Values	40	3.5	
Vehicle	Float/trailer—angle load	123	11.0	9.1–12.8
	Float/trailer—straight load	587	52.4	49.5–55.3
	Large truck—more than 3 horses	213	19.1	16.7–21.4
	Small truck—2 to 3 horses	128	11.4	9.5–13.2
	Use a commercial trucking company	68	6.1	4.6–7.5
	Total	1119	100	
	Missing Values	5	0.4	
Direction of travel	Head facing or angled to the front	842	77.5	75.0–79.9
	Head facing or angled to the rear	244	22.5	20.0–24.9
	Total	1086	100	
	Missing Values	38	3.4	
Sedation	Never	846	75.3	72.7–77.8
	Yes	277	24.7	22.1–27.2
	Total	1123	100	
	Missing Values	1	0.1	
Protective equipment	No	313	28.2	30.8–25.6
	Yes	797	71.8	74.4–69.2
	Total	1110	100	
	Missing Values	14	1.2	
Rugs	No	764	68.8	66.1–71.5
	Yes	346	31.2	28.4–33.9
	Total	1110	100	
	Missing Values	14	1.2	
Boots	No	530	47.7	44.7–50.6
	Yes	580	52.3	49.4–55.2
	Total	1110	100	
	Missing Values	14	1.2	
Training	Habituation	301	29.6	26.8–32.4
	None	238	23.4	20.8–26.0
	R−P+ ^1^	281	27.6	24.8–30.3
	R+ ^2^	45	4.4	3.1–5.6
	Self-loading	152	15.0	12.8–17.1
	Total	1017	100	
	Missing Values	107	9.5	
Whip at loading	No	949	84.7	82.5–86.8
	Yes	171	15.3	13.2–17.4
	Total	1120	100	
	Missing Values	4	0.35	
Bum rope at loading	No	893	79.4	77.0–81.7
	Yes	231	20.6	18.2–22.9
	Total	1124	100	
Food at loading	No	860	76.7	74.2–79.1
	Yes	261	23.3	20.8–25.7
	Total	1121	100	
	Missing Values	3	0.3	
Restraint	Cross tie	34	3.0	1.9–4.0
	I do not restrain my horse	124	11.1	9.2–12.9
	Tie up on a long rope	236	21.2	18.8–23.5
	Tie up on a short rope	721	64.7	61.9–67.5
	Total	1115	100	
	Missing Values	9	0.8	
Food *en route*	No	755	67.8	65.1–70.5
	Yes	358	32.2	29.4–34.9
	Total	1113	100	
	Missing Values	11	1.0	

^1^ R−P+: operant conditioning with a combination of negative reinforcement and positive punishment; ^2^ R+: operant conditioning using positive reinforcement.

**Table 4:** Results of univariate logistic regression analyses of associations between TRPBs and the explanatory variables, experience in horse handling, ability to recognize distress in horses, vehicle, type of training used to teach horses to load and travel, use of the whip or food at loading, type of restraint and feeding *en route*. Data were collected from an online survey on horse road transport in New Zealand (*n* = 1124) for movements occurring between 2015 and 2017 (OR: odds ratios; CI: confidence intervals; P^a^: LRT *P* value; P^b^: Wald test *P* value).

Variable	Category	TRPB—No *n* (%)	TRPB—Yes *n* (%)	OR	95%CI	P^a^	P^b^
Experience	>51	59 (85.5)	10 (14.5)	Ref			0.022
	41–50	125 (83.5)	24 (16.1)	1.13	0.50–2.52	0.766	
	31–40	206 (79.8)	52 (20.2)	1.49	0.71–3.10	0.301	
	21–30	169 (74.1)	59 (25.9)	2.06	0.99–4.28	0.060	
	11–20	195 (72.8)	73 (27.2)	2.21	1.00–4.54	0.036	
	6–10	79 (83.2)	16 (16.8)	1.19	0.50–2.82	0.692	
	1–5	33 (70.25)	14 (29.8)	2.50	1.1–6.25	0.049	
Distress ^1^	5—very high	477 (81.3)	110 (18.7)	Ref			0.010
	4—high	340 (73.45)	123 (26.6)	1.57	1.17–2.10	0.002	
	3,2—moderate, some	57 (78.1)	16 (18.7)	1.22	0.67–2.19	0.514	
Vehicle	Small truck—2 to 3 horses	113 (88.3)	15 (11.7)	Ref			0.003
	Float/trailer—straight load	434 (73.9)	153 (26.1)	2.66	1.50–4.69	<0.001	
	Float/trailer—angle load	101 (82.1)	22 (17.9)	1.64	0.80–3.33	0.171	
	Large truck—more than 3 horses	173 (81.2)	40 (18.8)	1.74	0.91–3.29	0.089	
	Use a commercial trucking company	50 (73.5)	18 (26.5)	2.71	1.26–5.80	0.010	
Training	Habituation	241 (80.1)	60 (19.9)	Ref			0.021
	Self-loading	123 (80.9)	29 (19.1)	0.95	0.57–1.55	0.829	
	None	190 (79.8)	48 (20.2)	1.01	0.66–1.55	0.946	
	R−P+ ^2^	197 (70.1)	84 (29.9)	1.71	1.17–2.50	0.006	
	R+ ^3^	34 (75.6)	11 (24.4)	1.30	0.62–2.70	0.484	
Whip at loading	No	758 (79.9)	191 (20.1)	Ref			<0.001
	Yes	113 (66.1)	58 (33.9)	2.04	1.43–2.90	<0.001	
Food at loading	No	694 (80.7)	166 (19.3)	Ref			
	Yes	178 (62.2)	83 (31.8)	1.95	1.43–2.65		
Restrain	Tied up on a short rope	581 (80.6)	140(19.4)	Ref			0.022
	I do not restrain my horse	93 (75.0)	31 (25.0)	1.38	0.88–2.15	0.153	
	Tied up on a long rope	171 (72.5)	65 (27.5)	1.57	1.12–2.21	0.008	
	Cross tied	23 (67.6)	11 (32.4)	1.98	0.94–4.10	0.069	
Food *en route*	No	619 (81.9)	136 (18.1)	Ref			<0.001
	Yes	249 (65.6)	109 (30.4)	1.99	1.48–2.66	<0.001	

^1^ No respondents chose option “1—none”; ^2^ R−P+: operant conditioning with a combination of negative reinforcement and positive punishment; ^3^ R+: operant conditioning using positive reinforcement.

**Table 5:** Results of multivariable regression analysis of associations between TRPBs and the explanatory variables, experience, industry sector, and type of involvement (amateur/professional). Data were collected from an online survey on horse road transport in New Zealand (*n* = 1124) for movements occurring between 2015 and 2017 (SE: standard error; OR: odds ratio; CI: confidence intervals; P^a^: LRT *P* value; P^b^: Wald test *P* value).

Variable	Category	Estimate	SE	OR	95%CI	P^a^	P^b^
Intercept		−2.5886	0.335	0.08	0.04–0.14	<0.001	
Vehicle	Small truck—2 to 3 horses	Ref					
	Large truck—more than 3 horses	0.862	0.355	2.37	1.21–4.91	0.015	
	Use a commercial trucking company	1.379	0.429	3.97	1.72–9.35	0.001	0.005
	Float/trailer—angle load	0.602	0.388	1.82	0.86–3.99	0.121	
	Float/trailer—straight load	1.034	0.318	2.81	1.56–5.47	0.001	
Training	Habituation	Ref					
	None	−0.124	0.227	0.88	0.56–1.38	0.585	
	Self-loading	−0.246	0.267	0.78	0.46–1.31	0.356	0.035
	R−P+ ^1^	0.416	0.206	1.52	1.01–2.27	0.043	
	R+ ^2^	−0.164	0.406	0.85	0.37–1.83	0.686	
Food	No	Ref					
	Yes	0.413	0.184	1.05	1.05–2.16	0.024	0.024
Whip	No	Ref					
	Yes	0.435	0.202	1.04	1.04–2.29	0.031	0.031
Food *en route*	No	Ref					
	Yes	0.635	0.165	1.89	1.37–2.61	<0.001	<0.001

^1^ R−P+: operant conditioning with a combination of negative reinforcement and positive punishment; ^2^ R+: operant conditioning using positive reinforcement.

## Supplementary Materials

The following are available online at http://www.mdpi.com/2076-2615/8/8/134/s1. Table S1: Univariate logistic regression Wald’s test *P* values calculated with TRPBs as outcome. Supplementary file: Survey used in the study.
