# Supplementary material for: Risk Factors for Transport-Related Problem Behaviors in Horses: A New Zealand Survey
Source: Animals (Basel). 2018 Aug 2;8(8):134. doi: 10.3390/ani8080134 (PMC6115720; doi:10.3390/ani8080134)
Supplement: Supplementary file 1 [file animals-08-00134-s001.pdf]

Supplementary file—Survey in the study

1. What is your sex?
- Male
- Female
- Other

2. What section of the horse industry or sport are you primarily involved in?
- Thoroughbred racing
- Standardbred racing
- Pony club
- Endurance
- Horse breeding
- Recreational riding
- Dressage
- Show jumping
- Eventing
- Reining
- Showing
- Polo
- Other (please specify) ____________________

3. What is your relationship with the horse(s)?
- Mostly amateur - involved with horses as a hobby or for recreational purposes
- Mostly professional - involved with horses as part of my job

4. How many years of experience do you have with handling horses?
- 1–5
- 6–10
- 11–20
- 21–30
- 31–40
- 41–50
- >51

5. Approximately how many horses are kept where you keep your horse(s)?
- 1–2
- 3–5
- 6–10
- 11–15
- 16–30
- >31

6. How do you grade your ability in identifying a horse in distress?
- 1—low
- 2—some
- 3—moderate
- 4—high
- 5—very high

7. How often do you transport your horse(s)?
- Daily

- 2 to 5 times a week
- Once weekly
- Fortnightly
- Monthly
- Less than once a month

8. What is the average kilometers traveled per trip when you have transported your horse(s) in the last year?

- 1–20
- 31–60
- 61–90
- 91–120
- 120–240
- >241

9. How do you usually transport your horse(s)?

- Small truck—2 to 3 horses
- Large truck—more than 3 horses
- Gooseneck
- Float/trailer—angle load
- Float/trailer—straight load
- Use a commercial trucking company

10. In your horse transport vehicle what direction is the horse facing?

- Head facing or angled to the front
- Head facing or angled to the rear
- Horse free and unrestrained

11. Do you use sedation or other product to calm your horse(s) prior to transportation?

- No
- Yes

12. Do you use protective equipment on your horse(s) during transportation? (tick all that apply)

- None
- Leg boots
- Leg bandages
- Poll protector
- Tail guard/bandage
- Neck rug
- Body rug
- Other (please specify) ____________________

13. Have you specifically trained your horse(s) for loading and traveling?

- No
- Yes

14. Please describe the method or the way in which you have trained your horse for loading and traveling:

15. Indicate below if you use any equipment or aids for loading your horse(s)? (tick all that apply)

- Whip
- Bum rope

- Food
- Halter and rope
- Bridle
- Stallion bit
- Load 'n' tie
- Other (please specify) ____________________

16. How do you restrain your horse(s) in the transport vehicle?

- I do not restrain my horse
- Tie up on a short rope
- Tie up on a long rope
- Cross tie
- Hobble
- Other (please specify) ____________________

17. Do you offer food to the horse(s) when travelling?

- No
- Yes—hay
- Yes—grain/complete feed

18. Has your horse(s) shown a behavioural problems associated with transport within the last 2 years? (e.g. anxiety, flight response, fight response, kicking, rearing, refusal to load)

- Yes (please describe) ____________________
- No

19. In which part of the transportation have you noticed the behavioural problem?

- Pre-loading (e.g. vocalisation, pawing, shaking)
- Loading (e.g. refusal to load, rearing, pulling back, kicking, pawing)
- Traveling (e.g. kicking, biting, scrambling, pawing)
- Unloading (e.g. rushing off, freezing)

Table S1. Univariate logistic regression Wald's test P values calculated with TRPBs as outcome.

**Predictive variable**	**P[a]**
Gender	0.701
Sector	0.211
Involvement	0.263
**Experience**	**0.022**
Number of horses	0.670
**Distress**	**0.010**
Journey frequency	0.325
Journey distance	0.926
**Vehicle**	**0.003**
Direction of travel	0.211
Sedation	0.152
Protective equipment	0.455
Rugs	0.212
Boots	0.569
**Training**	**0.021**
**Whip**	**<0.001**
Bum rope	0.224
**Food**	**<.001**
**Restraint**	**0.022**
**Food *en route***	**<0.001**
